# Drug candidates in clinical trials for Alzheimer’s disease

**DOI:** 10.1186/s12929-017-0355-7

**Published:** 2017-07-19

**Authors:** Shih-Ya Hung, Wen-Mei Fu

**Affiliations:** 1 Graduate Institute of Acupuncture Science, College of Chinese Medicine, China Medical University, Taichung, 40402 Taiwan; 2 Division of Colorectal Surgery, China Medical University Hospital, Taichung, 40447 Taiwan; 3 Pharmacological Institute, College of Medicine, National Taiwan University, No. 1, Sec. 1, Jen-Ai Road, Taipei, 10051 Taiwan

**Keywords:** Alzheimer’s disease, Clinical trials, Drug treatment, Neurodegenerative disease

## Abstract

Alzheimer’s disease (AD) is a major form of senile dementia, characterized by progressive memory and neuronal loss combined with cognitive impairment. AD is the most common neurodegenerative disease worldwide, affecting one-fifth of those aged over 85 years. Recent therapeutic approaches have been strongly influenced by five neuropathological hallmarks of AD: acetylcholine deficiency, glutamate excitotoxicity, extracellular deposition of amyloid-β (Aβ plague), formation of intraneuronal neurofibrillary tangles (NTFs), and neuroinflammation. The lowered concentrations of acetylcholine (ACh) in AD result in a progressive and significant loss of cognitive and behavioral function. Current AD medications, memantine and acetylcholinesterase inhibitors (AChEIs) alleviate some of these symptoms by enhancing cholinergic signaling, but they are not curative. Since 2003, no new drugs have been approved for the treatment of AD. This article focuses on the current research in clinical trials targeting the neuropathological findings of AD including acetylcholine response, glutamate transmission, Aβ clearance, tau protein deposits, and neuroinflammation. These investigations include acetylcholinesterase inhibitors, agonists and antagonists of neurotransmitter receptors, β-secretase (BACE) or γ-secretase inhibitors, vaccines or antibodies targeting Aβ clearance or tau protein, as well as anti-inflammation compounds. Ongoing Phase III clinical trials via passive immunotherapy against Aβ peptides (crenezumab, gantenerumab, and aducanumab) seem to be promising. Using small molecules blocking 5-HT_6_ serotonin receptor (intepirdine), inhibiting BACE activity (E2609, AZD3293, and verubecestat), or reducing tau aggregation (TRx0237) are also currently in Phase III clinical trials. We here systemically review the findings from recent clinical trials to provide a comprehensive review of novel therapeutic compounds in the treatment and prevention of AD.

## Background

### Epidemiology and pathogenesis of Alzheimer’s disease

Alzheimer’s disease (AD) was first described and diagnosed by Dr. Alois Alzheimer in 1906 [1]. According to World Health Organization (WHO), AD is the most common cause of dementia, accounting for as many as 60 ~ 70% of senile dementia cases and affecting 47.5 million people worldwide in 2015 [2]. The median survival time after the onset of dementia ranges from 3.3 to 11.7 years [3]. AD is characterized as a severe, chronic and progressive neurodegenerative and incurable disorder, associated with memory loss and cognition impairment accompanied by abnormal behavior and personality changes [4]. Age is a risk factor for AD, which is the most common cause of dementia affecting persons aged over 65 years [5]. Over 95% of all AD cases are diagnosed as having late-onset AD and are aged 65 years and over; only 1 ~ 5% of all cases are early-onset AD [4]. Globally, the incidence rate for AD doubles every five years after the age of 65. As the average age of the population increases, the number of cases of AD is expected to more than triple by 2050, reaching over 115 million [6]. The direct societal cost of AD is second only to cancer care. In the US alone, an estimated $172 billion is spent annually on AD-related health-care costs [7].

**Fig. 1:**
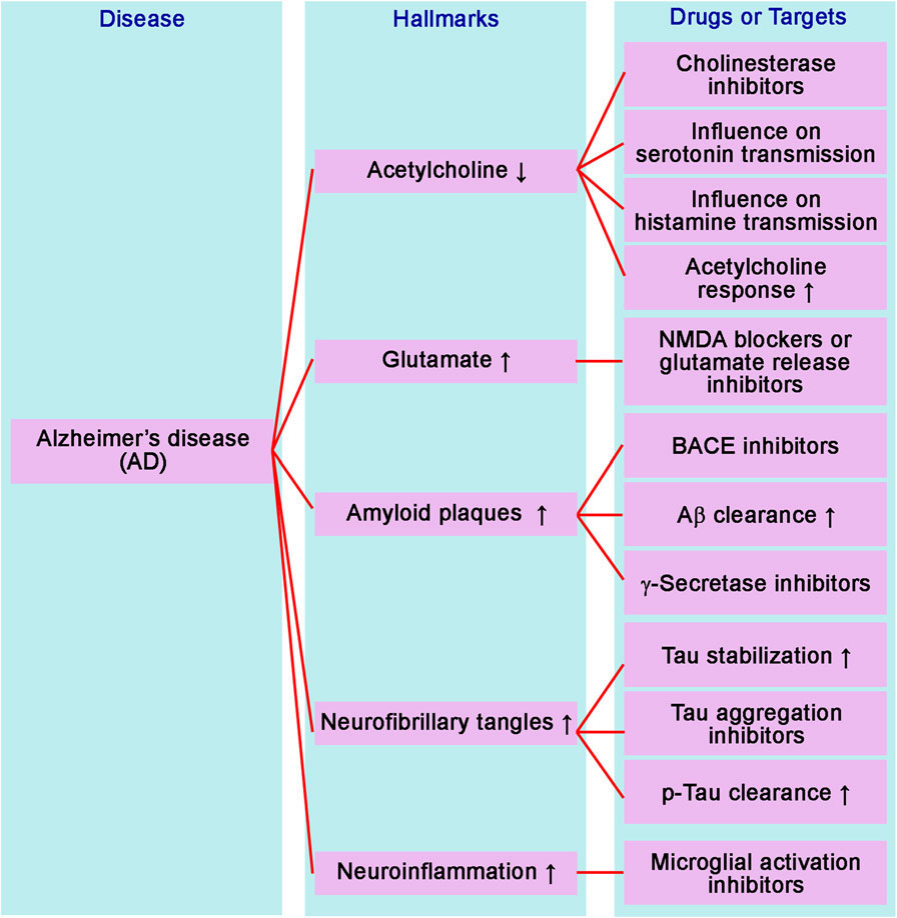
Classification of therapeutic drugs or targets in the treatment of Alzheimer’s disease according to neuropathological hallmarks

AD is characterized by neuronal death, which usually correlates with the appearance of key neuropathological changes, including acetylcholine deficiency, glutamate excitotoxicity, extracellular deposition of β-amyloid (Aβ plaques), intracellular neurofibrillary tangles by hyperphosphorylated tau protein deposits, neuroinflammation, and widespread neuronal loss [4, 8]. The role of Aβ and tau proteins in the pathophysiology of AD remains unclear. Different theories suggest that inflammation, accumulation of reactive oxygen species (ROS), mitochondrial damage, genetic factors, cerebrovascular disease, traumatic brain injury, age-related loss of sex steroid hormones in both women and man, are some of the established risk factors considered to be promising targets for drug discovery in the treatment of AD [7, 9, 10]. We have classified therapeutic drugs and targets in the treatment of AD according to the neuropathological hallmarks of AD (Fig. 1).

## Lack of acetylcholine in Alzheimer’s disease

In AD, the loss of cholinergic tone and acetylcholine levels in the brain is hypothesized to be responsible for the gradual cognitive decline.

### Enhancement of the acetylcholine response by acetylcholinesterase inhibitors

In 1976, Davies and Maloney were the first to hypothesize that selective loss of central cholinergic neurons in AD plays a key role in its pathophysiology [11]. The release in 1993 of tacrine, the first reversible acetylcholinesterase inhibitor (AChEI) to reach the market for the treatment of AD, was withdrawn soon after because of reports of liver toxicity. Three other cholinesterase inhibitors- donepezil, galantamine, and rivastigmine are currently used in the treatment of AD to reduce the activity of acetylcholinesterase. These agents do not delay the progression of dementia but temporarily slow the loss of cognitive function.

### Enhancement of the acetylcholine response using 5-HT_6_ receptor antagonists

The serotonergic neurotransmitter system is impaired as AD develops and progresses; modulation of this pathway is therefore considered to be of therapeutic value [12]. Serotonin (5-HT) activates specific 5-HT receptors, consisting of seven distinct classes (5-HT_1_ to 5-HT_7_) based on their structural and function characteristics. The 5-HT_6_ receptor is expressed primarily in brain areas involved in learning and memory processes – the cortex and hippocampus. 5-HT_6_ receptor antagonists are thought to enhance cholinergic neurotransmission [12]. Idalopirdine (Lu AE58054) is an orally available 5-HT_6_ antagonist, that showed promising efficacy and safety data in Phase II trials (Clinical Trial Identifier: NCT01019421). Although idalopirdine is safe and well tolerated as an adjunctive therapy to donepezil (AChEI) in patients with mild-to-moderate AD, however, idalopirdine did not meet its primary efficacy endpoint versus placebo in recent two phase III trials [13] (Clinical Trial Identifier: NCT02006641 and NCT02006654) (Table 1). Intepirdine (RVT-101) is another 5-HT_6_ antagonist that is currently in Phase III clinical trials in patients with mild-to-moderate AD already on donepezil therapy (Clinical Trial Identifier: NCT02585934 and NCT02586909) (Table 1). Analysis of data from Phase II evaluation of treatment with intepirdine indicates that addition of this treatment to donepezil may improve the cognition and function of patients with mild-to-moderate AD [14] (Clinical Trial Identifier: NCT02910102).

**Table 1:** Update of selected anti-Alzheimer’s disease drugs in clinical trials (updated in June 2017)

Target	Drug name	Therapy type	Trial status	Reasons for Discontinuation	Company	Clinical Trial Identifier	References
Serotoninergic (5-HT_6_ receptor antagonist)	Idalopirdine	Small molecule	Phase III Discontinued in 2017	No clinical efficacy	H. Lundbeck, Otsuka Pharmaceutical Co., Ltd.	NCT01019421 NCT02006641 NCT02006654	[13]
	Intepirdine	Small molecule	Phase II/III	Not applicable	Axovant Sciences Ltd.	NCT02910102 NCT02585934 NCT02586909	[14]
Histaminergic (H_3_ receptor antagonist)	ABT-288	Small molecule	Phase II Discontinued in 2011	No clinical efficacy	AbbVie	NCT01018875	[16]
	GSK239512	Small molecule	Phase II Discontinued in 2012	No improvements in memory test	GlaxoSmithKline (GSK)	NCT01009255	[17]
	SUVN-G3031	Small molecule	Phase I	Not applicable	Suven Life Sciences Ltd	NCT02342041	
Acetylcholine response ↑ (α7nAChR agonist)	Encenicline	Small molecule	Phase III Discontinued in 2015	Adverse effects: gastrointestinal side effect	FORUM Pharmaceuticals Inc., Mitsubishi Tanabe Pharma	NCT01969136 NCT01969123	[23, 24]
Glutaminergic	Riluzole	Small molecule	Phase II	Not applicable	Sanofi	NCT01703117	[29–32]
BACE inhibitor	BI 1181181	Small molecule	Phase I Discontinued in 2015	Low oral bioavailability and low blood-brain barrier penetration	Boehringer Ingelheim, Vitae Pharmaceuticals	NCT02044406 NCT02106247 NCT02254161	
	RG7129	Small molecule	Phase I Discontinued in 2013	Liver toxicity	Roche	NCT01664143 NCT01592331	
	LY2811376	Small molecule	Phase I Discontinued In 2008	Liver toxicity	Eli Lilly & Co.	NCT00838084	
	LY2886721	Small molecule	Phase II Discontinued In 2013	Liver toxicity	Eli Lilly & Co.	NCT01561430	[36]
	E2609	Small molecule	Phase III	Not applicable	Biogen, Eisai Co., Ltd.	NCT03036280 NCT02956486	
	AZD3293	Small molecule	Phase III	Not applicable	AstraZeneca, Eli Lilly & Co.	NCT02783573	
	CNP520	Small molecule	Phase II/III	Not applicable	Amgen, Inc., Novartis Pharmaceuticals Corporation	NCT02576639 NCT02565511	
	JNJ-54861911	Small molecule	Phase II/III	Not applicable	Janssen, Shionogi Pharma	NCT02406027 NCT02569398	
	Verubecestat	Small molecule	Phase III	Not applicable	Merck	NCT01953601	[37]
γ-Secretase inhibitor	Semagacestat	Small molecule	Phase III Discontinued in 2012	No clinical efficacy and adverse effects: skin cancer and infections	Eli Lilly & Co.	NCT00762411 NCT00594568 NCT01035138	[40]
	Avagacestat	Small molecule	Phase II Discontinued in 2012	Adverse effects: cerebral microbleeds, glycosuria, and nonmelanoma skin cancer	Bristol-Myers Squibb	NCT00890890	
	EVP-0962	Small molecule	Phase II Discontinued in 2016	Not applicable	FORUM Pharmaceuticals Inc.	NCT01661673	[41]
	NIC5-15	Small molecule	Phase II	Not applicable	Humanetics Pharmaceuticals Corporation	NCT00470418 NCT01928420	
Aβ clearance	AN-1792	Active immunotherapy (Aβ_1-42_ peptides)	Phase II Discontinued in 2002	Adverse effects: meningoencephalitis	Janssen, Pfizer	NCT00021723	[44]
	CAD106	Active immunotherapy (Aβ_1-6_ peptides)	Phase II/III	Not applicable	Novartis Pharmaceuticals Corporation	NCT01097096 NCT02565511	[47]
	ACC-001	Active immunotherapy (Aβ_1-4_ peptides)	Phase II Discontinued in 2013	Adverse effects: strong autoimmune response	Janssen	NCT01238991 NCT00479557 NCT00498602	[48]
	Affitope AD02	Active immunotherapy (6 a.a. peptides mimic Aβ_1-42_ N-terminus)	Phase II-discontinued in 2014	Not applicable	AFFiRiS AG	NCT01117818	[49]
	Bapineuzumab	Passive immunotherapy (against Aβ N-terminal)	Phase III- discontinued in 2012	No clinical efficacy	Janssen, Pfizer	NCT00667810 NCT00676143	[50]
	AAB-003	Passive immunotherapy	Phase I	Not applicable	Janssen, Pfizer	NCT01193608 NCT01369225	
	GSK933776	Passive immunotherapy (against Aβ N-terminal)	Phase I Discontinued in 2012	No clinical benefit	GlaxoSmithKline (GSK)	NCT00459550 NCT01424436	
	Solanezumab	Passive immunotherapy (against Aβ_16-24_)	Phase III Discontinued in 2016	Missed primary endpoint	Eli Lilly & Co.	NCT01127633 NCT01900665	[51–53]
	Crenezumab	Passive immunotherapy against Aβ)	Phase III	Not applicable	AC Immune SA, Genentech, Hoffmann-La Roche	NCT02670083	
	Gantenerumab	Passive immunotherapy (against Aβ_3-12_ & Aβ_18-27_)	Phase III	Not applicable	Chugai Pharmaceutical Co., Ltd., Hoffmann-La Roche	NCT02051608 NCT01900665	
	BAN2401	Passive immunotherapy (against large soluble Aβ protofibrils)	Phase II	Not applicable	Biogen, Eisai Co., Ltd.	NCT01767311	
	Aducanumab	Passive immunotherapy (against aggregated Aβ)	Phase III	Not applicable	Biogen	NCT02477800 NCT02484547	[54]
Tau stabilization	Epothilone D	Small molecule	Phase I Discontinued in 2013	Not applicable	Bristol-Myers Squibb	Not available	
	TPI 287	Small molecule	Phase I	Not applicable	Cortice Biosciences	NCT01966666	
Tau aggregation inhibitor	Rember™	Small molecule	Phase II Discontinued in 2007	Adverse effects: diarrhea, urinary urgency, and painful urination, etc.	TauRx Therapeutics Ltd	NCT00515333 NCT00684944	[59, 60]
	TRx0237	Small molecule	Phase III	Not applicable	TauRx Therapeutics Ltd	NCT01689233 NCT01689246 NCT01626378	[61]
p-Tau clearance	AADvac-1	Active immunotherapy (synthetic peptide truncated and misfolded tau)	Phase II	Not applicable	Axon Neuroscience SE	NCT01850238 NCT02031198 NCT02579252	[61, 62]
	ACI-35	Active immunotherapy (Human protein tau sequence 393 to 408 of longest tau isoform phosphorylated at S396 and S404)	Phase I	Not applicable	AC Immune SA, Janssen	Main ID in the WHO International Clinical Trials Registry Platform: ISRCTN13033912	[61–63]
Microglial activation inhibitor	Alzhemed™	Small molecule	Phase III Discontinued in 2007	No clinical efficacy	Neurochem, Inc.	Not available	[64]
	Azeliragon	Small molecule	Phase III	Not applicable	Pfizer, TransTech Pharma, Inc., vTv Therapeutics LLC	NCT02080364	
	Ibuprofen	Small molecule	Phase IV Discontinued in 2005	No clinical efficacy	Not applicable	Not available	[65]
	Flurizan™	Small molecule	Phase III Discontinued in 2008	No clinical efficacy	Myriad Genetics & Laboratories	NCT00322036	
	CHF 5074	Small molecule	Phase II	Not applicable	CereSpir™ Incorporated, Chiesi Pharmaceuticals Inc.	NCT01421056	[66]

### Enhancement of the acetylcholine response using H_3_ receptor antagonists

Histamine H_3_ receptors are widely distributed throughout the CNS. Blockade of this receptor augments the presynaptic release of both histamine and other neurotransmitters including acetylcholine. Several histamine H_3_ antagonists have entered therapeutic programs for cognition disorders [15], including ABT-288, GSK239512, and SUVN-G3031 (Table 1). A Phase II trial of ABT-288 in patients with mild-to-moderate AD as adjunct treatment on stable donepezil was ended due to lack of clinical efficacy [16] (Clinical Trial Identifier: NCT01018875). Similarly, GSK239512 was discontinued in a Phase II study because of lack of improvement in memory testing in patients with mild-to-moderate AD (Clinical Trial Identifier: NCT01009255), suggesting that H_3_ receptor antagonists are not effective in treating cognitive dysfunction in AD [17]. SUVN-G3031 is an orally active H_3_ receptor antagonist, which is currently undergoing Phase I investigation evaluating the safety, tolerability, and pharmacokinetics study in healthy volunteers (Clinical Trial Identifier: NCT02342041).

### Enhancement of the acetylcholine response by α7 nicotinic acetylcholine receptor (α7nAChR) agonists

It is well established that acetylcholine neurotransmission plays a crucial role in learning and memory. Current medication is aimed at enhancing cholinergic signaling for treating cognitive deficits and memory impairment in neurodegenerative disorders, including AD. The nicotinic acetylcholine receptor family (nAChR) and the muscarinic acetylcholine receptor family (mAChR) are targeted by acetylcholine in the brain [18]. The alkaloid galantamine, used for the treatment of mild-to-severe dementia, has shown activity in modulating the nicotinic cholinergic receptors on cholinergic neurons to increase acetylcholine release [19]. α7nAChR belongs to the family of ligand-gated ion channels and is expressed in key brain regions (e.g. prefrontal and frontal cortices, hippocampus) [20]. α7nAChR is involved in essential cognitive functions such as memory, thinking, comprehension, the capacity to learn, calculate, orientate, language abilities, and judgment [20]. Notably, α7nAChR acts as a carrier to bind with extracellular Aβ, which further inhibits Aβ-induced neurotoxicity via autophagic degradation, an important step in Aβ detoxification [21, 22]. Encenicline (EVP-6124, MT-4666), a partial selective agonist of α7nAChR, has been developed for the treatment of cognitive deficits in AD and schizophrenia and AD (Table 1). Clinical study findings show that encenicline also can act as a co-agonist with acetylcholine to enhance cognition [23]. In 2015, the U.S. FDA imposed a clinical hold on encenicline following reports of gastrointestinal side effects in two Phase III Alzheimer studies [24] (Clinical Trial Identifier: NCT01969136 and NCT01969123).

## Glutamate transmission in Alzheimer’s disease

### Inhibition of glutamate cytotoxicity by NMDA receptor antagonists or glutamate release inhibitors

Excitotoxicity resulting from excessive activation of *N*-methyl-D-aspartate (NMDA) receptors may enhance the localized vulnerability of neurons in a manner consistent with AD neuropathology [25]. Memantine (Akatinol®) is the only NMDA receptor antagonist used clinically in the treatment of AD. Since 1989, memantine has been available in Germany for the treatment of dementia. Memantine also protects neurons from glutamine-mediated excitotoxicity [26]. Memantine is the last AD drug approved by the U.S. FDA in 2003. Memantine also cannot prevent neuronal loss, worsening of dementia or modify the disease progression. Despite never receiving formal approval for the early stages of AD, memantine is frequently prescribed at this stage. Its clinical usefulness in mild AD is controversial, with conflicting reports from meta-analyses [27, 28]. The sodium channel blocker, riluzole, is approved by the U.S. FDA as a disease-modifying drug for amyotrophic lateral sclerosis that lowers extracellular glutamate levels [29] (Table 1). It is known that riluzole also inhibits presynaptic glutamate release and enhances glutamate transporter activity [30, 31]. In tau-P301L transgenic mice (an AD animal model), riluzole treatment increases glutamate reuptake and decreases glutamate release in hippocampus [32]. Riluzole also recues TauP301L-mediated reductions in PSD-95 expression (a marker of the excitatory synapses) in the brain and reduced total levels of tau, as well as the pathological phosphorylation and conformational changes in tau associated with the P301L mutation [32]. These findings suggest a new clinically applicable therapeutic approach for patients at risk for the development of AD. Riluzole is currently being investigated for beneficial cognitive effects in a Phase II clinical trial involving patients with mild AD already receiving donepezil (Clinical Trial Identifier: NCT01703117).

## Aβ pathway and the amyloid hypothesis in Alzheimer’s disease

Aβ is derived from amyloid precursor protein (APP) in a two-step proteolysis reaction by two membrane-bound enzyme complexes, β-secretase (BACE) and γ-secretase [8]. The amyloid (or Aβ) hypothesis contends that the deposition of the Aβ peptide in the brain is the primary cause of AD pathology [33]. This hypothesis is guiding the development of potential treatments. Compelling preclinical and emerging clinical evidence supports a key role for Aβ dyshomeostasis in initiating AD [33]. Therefore, β- and γ-secretase have attracted strong interest as potential targets for drugs and monoclonal antibodies that might reduce Aβ deposition [8].

### Inhibition of Aβ production by β-secretase (BACE) inhibitors

According to the Aβ hypothesis, Aβ-related toxicity is the primary cause of synaptic dysfunction and subsequent neurodegeneration characteristic of AD [34]. β-secretase is a type 1 transmembrane aspartic acid protease related to the pepsin family. The enzyme BACE1 (β-site APP cleaving enzyme 1) plays a crucial role in the generation of Aβ and has therefore been pursued as a small molecular drug target to modulate Aβ production. The rationale of BACE inhibition is that it represents a way of interfering upstream in the amyloid cascade. BACE1 mRNA is increased in both AD patients and animal models of disease [35]. The orally active BACE1 inhibitor, BI 1181181, is the first generation of BACE1 inhibitor failed in Phase I trials because of low oral bioavailability and low blood-brain barrier penetration (Clinical Trial Identifier: NCT02044406, NCT02106247, and NCT02254161) (Table 1). Subsequent, second-generation BACE1 inhibitors, RG7129 (Phase I; Clinical Trial Identifier: NCT01664143 and NCT01592331), LY2811376 (Phase I; Clinical Trial Identifier: NCT00838084), and LY2886721 (Phase II; Clinical Trial Identifier: NCT01561430) also failed in clinical trials because of liver toxicity [36] (Table 1). However, third-generation BACE1 inhibitors including E2609 (Phase III; Clinical Trial Identifier: NCT03036280 and NCT02956486), AZD3293 (Phase III; Clinical Trial Identifier: NCT02783573), CNP520 (Phase II/III; Clinical Trial Identifier: NCT02576639 and NCT02565511), JNJ-54861911 (Phase II/III; Clinical Trial Identifier: NCT02406027 and NCT02569398) have shown satisfactory pharmacokinetics and encouraging clinical data in ongoing studies. Although verubecestat (MK-8931) reduces CNS Aβ in animal models and in AD patients [37], however, Merck has announced in February 2017 that they will stop the clinical study in mild-to-moderate AD patients because of lack of efficacy [38]. APECS trial in people with prodromal AD still continues (Clinical Trial Identifier: NCT01953601).

### Inhibition of Aβ production by γ-secretase inhibitors or modulators

γ-secretase is a protease complex containing four subunits: nicastrin (NCSTN), presenilin (PEN-1), anterior pharynx-defective 1 (APH-1), and presenilin enhancer 2 (PEN-2) [39]. Each subunit is regarded as a potential therapeutic for modulating Aβ production or increasing Aβ clearance. However, the γ-secretase inhibitor semagacestat failed to achieve the primary endpoints in Phase III clinical trials because of worsening symptoms in some patients (NCT00762411, NCT00594568, and NCT01035138) [40]. Avagacestat was discontinued in a Phase II clinical trial (Clinical Trial Identifier: NCT00890890), after causing serious adverse events such as cerebral microbleeds, dose-dependent glycosuria, and nonmelanoma skin cancer (Table 1). The orally available, small molecule, selective γ-secretase modulator EVP-0962 reduces Aβ_1-42_ production by shifting the APP cleavage toward the production of shorter and less toxic Aβ peptides, without affecting Notch cleavage [41]. Although EVP-0962 showed promise in transgenic Alzheimer’s models, reducing Aβ_1-42_ peptide levels, decreasing amyloid plaque build-up, reversing behavioral deficits and reducing brain inflammation associated with AD, EVP-0962 was discontinued in Phase II clinical trials in the USA (Clinical Trial Identifier: NCT01661673) (Table 1). NIC5-15, pinitol, is a naturally occurring cyclic sugar alcohol that modulates γ-secretase to reduce Aβ production (Table 1). NIC5-15 is currently in Phase II trials in the treatment of AD (Clinical Trial Identifier: NCT00470418 and NCT01928420).

### Enhancement of Aβ clearance by active immunotherapy

Immunotherapy has been under investigation as a therapeutic approach to AD via active and passive vaccines against Aβ; however, translating these results safely and effectively into humans has been challenging [42]. The first report of immunotherapy treatment was published in 1999 [43]. An active immunization study using the AN-1792 Alzheimer vaccine (a synthetic full-length Aβ_1-42_ peptide with QS-21 adjuvant) in patients with mild-to-moderate AD was discontinued in Phase II because of severe meningoencephalitis developing in 6% of the patients [44] (Clinical Trial Identifier: NCT00021723) (Table 1). Next-generation vaccines are working to target more specific epitopes to induce a more controlled immune response [45, 46]. An active vaccination strategy that aims to elicit a strong antibody response while avoiding inflammatory T cell activation is CAD106, which uses the Aβ_1-6_ peptide in an immunogenic sequence to serve as a B-cell epitope and avoid a T-cell response [47]. CAD106 is in Phase II/III in cognitively unimpaired individuals with 2 ApoE4 genes. (Clinical Trial Identifier: NCT01097096 and NCT02565511) (Table 1). ACC-001 (vanutide cridificar) is an N-terminal Aβ_1-7_ amino acid peptide fragment linked to inactivated diphtheria toxin as the carrier [48]. ACC-001 was discontinued in Phase II trials because of a strong autoimmune response (Clinical Trial Identifier: NCT01238991, NCT00479557, and NCT00498602) (Table 1). Affitope AD02 contains six amino acids that mimics the N-terminus of Aβ and is in Phase II clinical investigation [49] (Clinical Trial Identifier: NCT01117818).

### Enhancement of Aβ clearance by passive immunotherapy

For the amyloid-based approach, passive anti-Aβ immunization is the most advanced strategy for treating AD. Bapineuzumab, a humanized form of murine monoclonal antibody that binds the N-terminal epitope Aβ, was terminated in two Phase III trials because of a lack of efficacy in patients with mild-to-moderate AD [50] (Clinical Trial Identifier: NCT00667810 and NCT00676143) (Table 1). AAB-003, a derivative of bapineuzumab, completed in Phase I trials in 2014 (Clinical Trial Identifier: NCT01193608 and NCT01369225) (Table 1). GSK933776 is a humanized mouse IgG_1_ monoclonal antibody directed against the N-terminus of the Aβ peptide. This antibody has failed to show any clinical benefit (Clinical Trial Identifier: NCT00459550 and NCT01424436) (Table 1). Solanezumab is a monoclonal antibody directed against Aβ_16-24_ (Table 1). It recognizes soluble monomeric but not fibrillary Aβ. However, in Phase III trial involving 2100 patients with mild AD, solanezumab failed to meet the primary endpoint [51, 52] (Clinical Trial Identifier: NCT01900665 and NCT01127633). Now, solanezumab is being tested in a prevention study in asymptomatic older subjects, who have positive positron emission tomography (PET) scans for brain amyloid deposits [53]. Crenezumab recognizes oligomeric and fibrillar Aβ species and amyloid plaques with high affinity, and monomeric Aβ with low affinity (Table 1). Crenezumab began enrolling patients with prodromal-to-mild AD for a Phase III study in 2016, which is expected to run until 2020 (Clinical Trial Identifier: NCT02670083). Gantenerumab is a conformational antibody against Aβ fibrils (Table 1), being tested in patients with mild AD in Phase III clinical trials (Clinical Trial Identifier: NCT02051608 and NCT01900665). BAN2401 binds to large soluble Aβ protofibrils and is thought to lead to Aβ clearance or neutralize Aβ toxicity (Table 1). It is currently in a Phase II trial in subjects with early AD (Clinical Trial Identifier: NCT01767311). Aducanumab, targeting aggregated but not monomer Aβ (Table 1), is currently in Phase III trials in patients with early AD (Clinical Trial Identifier: NCT02477800 and NCT02484547). Aducanumab can significantly reduce brain Aβ plaques in AD patients [54]. To date, no antibody-based immunotherapy targeting Aβ clearance has been on market.

## Tau pathway and neurofibrillary tangles (NFTs) hypothesis in AD

NFTs, or aggregation of hyperphosphorylated tau protein are a key feature of AD. Tau is a 50 ~ 75 kDa protein with six different splice variants (0N3R, 1N3R, 2N3R, 0N4R, 1N4R, and 2N4R) [55, 56]. The tau pathway is primarily an intracellular pathway that affects neurons. Soluble tau aggregates subsequently assemble and form paired helical filaments that go on to form intracellular NFTs, a process that causes cell death [57]. The molecular event leading to NFTs formation and neurodegeneration expands remains unclear. Therapeutic strategies that target the tau pathway focus mainly on limiting pathological tau phosphorylation that drives early aggregation [57]. Given the repeated failures of trials targeting the Aβ pathway in mild or moderate AD [53], there is increasing interest in the possibility that tau-targeted compounds could have therapeutic utility in AD, particularly tau aggregation inhibitors. At present, therapies targeting tau aim to reduce, stabilize, or prevent aggregation or hyperphosphorylation of the protein [57].

### Targeting tau aggregates

According to tauopathies, hyperphosphorylated tau is the form of the protein found in the paired helical filaments that make up NFTs. Pathological tau can cause disturbances of microtubules leading to neuronal degeneration; aggregated tau is cytotoxic.

### Enhancement of microtubule stabilization by tau stabilizers

Among tau-based anti-AD drugs, several microtubule stabilizing agents have been tested and these studies have provided proof of concept that compounds with the ability to stabilize microtubules may have therapeutic potential for the treatment of AD and other neurodegenerative diseases [58]. Epothilone D is a small molecule microtubule stabilizer; a Phase I trial study of epothilone D for AD ended in 2013 and further evaluation subsequently discontinued (Table 1). TPI 287 is a tubulin-binding and microtubule-stabilizing drug in Phase I trials in patients with mild-to-moderate AD, which is set to run until 2017 (Clinical Trial Identifier: NCT01966666) (Table 1). Tau stabilizers fail to reach the clinic, due to toxic side effects (paclitaxel) or have been discontinued for AD (epothilone D), or are in Phase I clinical development (TPI 287) for mild-to-moderate AD.

### Prevention of tau aggregation by tau aggregation inhibitors

Prevention of aggregation regardless of phosphorylation or other tau modification is another therapeutic approach. Derivatives of methylene blue have been shown to disrupt the aggregation of tau, such as Rember™ (a first-generation tau protein aggregation inhibitor), which showed some improvement in AD-related symptoms but failed in Phase II because of emergent side effects including diarrhea, urinary urgency, painful urination, dizziness, and falls [59, 60] (Clinical Trial Identifier: NCT00515333 and NCT00684944) (Table 1). TRx0237 (LMTM) is a second-generation tau protein aggregation inhibitor currently in three Phase III trials. Results of the first Phase III trial in patients with mild AD await presentation (Clinical Trial Identifier: NCT01689233). The second Phase III trial, in patients with mild-to-moderate AD, produced negative results [61] (Clinical Trial Identifier: NCT01689246); the third trial in behavioral variant frontotemporal dementia failed to achieve its co-primary endpoints [61] (Clinical Trial Identifier: NCT01626378).

### Enhancement of phosphorylated-tau clearance by active immunotherapy

High-affinity antibodies against phosphorylated-tau from active immunization of phosphorylated-tau is one approach, such as AADvac-1, which contains synthetic tau_294-305_ peptides, and also ACI-35, which contains phosphorylated S396 and S404 tau fragments; AADvac-1 is in Phase II trials and ACI-35 is in Phase I trials [57] (Table 1). AADvac1 contains a synthetic protein that corresponds to a naturally occurring truncated and misfolded tau protein coupled to keyhole limpet hemocyanin and aluminum hydroxide adjuvant [62]. The vaccine has undergone a Phase I trial in patients with mild-to-moderate AD (ClinicalTrials.gov Identifier: NCT01850238), which is now in follow-up (ClinicalTrials.gov Identifier: NCT02031198) and is considering advancement to Phase II trials (ClinicalTrials.gov Identifier: NCT02579252). ACI-35 is a liposomal-based 16-amino acid tetrapalmitoylated phosphor-tau peptide (a synthetic peptide of human protein tau sequence 393 to 408 of the longest tau isoform and is phosphorylated at S396 and S404) [62, 63]. It is a liposomal vaccine and Phase I trials have begun for ACI-35 (main ID in the WHO International Clinical Trials Registry Platform: ISRCTN13033912).

## Neuroinflammation in Alzheimer’s disease

After Aβ plaques and tau NFTs, neuroinflammation is one of the neuropathological correlates of AD. AD brain tissue presents clear evidence of astrogliosis and other inflammation-related signs surrounding amyloid plaques. Preclinical and clinical studies indicate a strong association between microglia hyperactivation in amyloid plaques formation and AD progression.

### Inhibition of neuroinflamation by microglial activation inhibitors

The small molecule microglial activation inhibitor, Alzhemed™ (Tramiprosate), failed to show clinical efficacy in a Phase III clinical trial in 2007 [64]. Azeliragon is an oral, small molecule inhibitor that has been associated with reductions in brain Aβ levels and improved cognitive performances; azeliragon is currently in a Phase III trial in patients with mild AD (Clinical Trial Identifier: NCT02080364). Ibuprofen is a non-steroidal anti-inflammatory drug (NSAID) that inhibits cyclooxygenase activity and further reduces inflammation through reduced prostaglandin synthesis. Clinical trials confirmed that ibuprofen had no significant cognitive benefit for the treatment of AD and was associated with nausea and vomiting [65]. Flurizan™ (r-flurbiprofen) is another NSAID (structurally and pharmacologically related to ibuprofen) that is used to treat several inflammatory conditions. Flurizan™ failed in a Phase III trial to make any difference to primary outcomes in mild AD (Clinical Trial Identifier: NCT00322036). CHF 5074, a microglia modulator, specifically drives the expression of microglia M2 markers in young Tg2576 hippocampus [66]. CHF 5074 is still in phase II for mild cognitive impairment due to Alzheimer’s disease (Clinical Trial Identifier: NCT01421056) (Table 1).

## New targets or therapeutic approaches in other mechanisms

### Intravenous immunoglobulin (IVIG)

IVIG is a naturally occurring antibody derived from the blood plasma of healthy donors which is being used for the treatment of autoimmune and inflammatory diseases [67]. The rationale for IVIG in AD treatment is that IVIG can direct against Aβ [68]. Although IVIG shows safe, well tolerated, anti-amyloid and immune modulatory properties in patients with AD, later Phase III trials indicate that IVIG exerts effects on cognition or function in patients with mild-to-moderate AD [69] (Clinical Trial Identifier: NCT00818662).

### Nasal insulin

Insulin is a hormone for Type 1 diabetes treatment, the rationale for AD treatment is that brain areas affected in AD patients show decrease in the concentration of insulin and increase in the number of insulin receptors [70]. In addition, intranasal insulin improves cognition and modulates β-amyloid in early AD [71]. The Phase II/III trials are currently ongoing to examine whether intranasal insulin by nasal spray improves memory in patients with mild cognitive impairment or AD (Clinical Trial Identifier: NCT01767909).

### Calcium channel blocker

Epidemiological evidence shows that chronic high blood pressure increases the risk for dementia and nilvadipine is a calcium channel blocker for hypertension treatment. In the transgenic mouse model of AD, nilvadipine reduces brain Aβ levels and improves Aβ clearance across the blood-brain barrier [72]. Now, the Phase III trial of nilvadipine has been completed in patients with mild-to-moderate AD and results await presentation [73] (Clinical Trial Identifier: NCT02017340).

## Conclusions

Current drug treatment for AD patients, essentially symptomatic, is based on three cholinesterase inhibitors (rivastigmine, donepezil and galantamine) and memantine, affecting the glutamatergic system. These drugs do not represent a cure, as they do not arrest the progression of dementia, but rather, they lead to a temporary slowdown in the loss of cognitive function by decreasing cholinesterase activity, resulting in higher ACh levels and improved brain function. Over two hundred compounds have reached Phase II clinical trials since 2003, but no new drugs have been approved for the treatment of AD [4, 10]. Most Phase II clinical trials ending with a positive outcome do not succeed in Phase III, often due to serious adverse effects or lack of therapeutic efficacy [10]. Other challenges facing drug development in AD include a lack of validated objective diagnostic criteria and robust biological markers of disease that might be useful as clinical endpoints and efficacy standards. Despite decades of study efforts to develop therapies, there is no effective therapy available to cure AD or significantly inhibits the progression of AD symptoms.

The development of new effective drugs targeting the CNS typically involves a difficult and time-consuming process, accompanied by a very high failure rate. AD is by far the most common dementia in late life. It is currently estimated that 48 million people worldwide have dementia with an estimated global cost of dementia care of US$818 billion in 2015, an increase of 35% since 2010 [74]. By 2030 it is estimated that there will be 74.7 million people with dementia, and the cost of caring for these individuals could rise to some US$2 trillion [75]. In the absence of effective therapies, the estimated number of people with dementia will reach 115 to 131.5 million by 2050 [6, 75]. Novel therapies and new targets are urgently needed for developing more effective drugs for the treatment of AD patients.

## References

[1] Hippius H, Neundorfer G (2003). The discovery of Alzheimer’s disease. Dialogues Clin Neurosci.

[2] Dementia Fact sheet N°362 [http://www.who.int/mediacentre/factsheets/fs362/en/].

[3] Todd S, Barr S, Roberts M, Passmore AP (2013). Survival in dementia and predictors of mortality: a review. Int J Geriatr Psychiatry.

[4] Godyn J, Jonczyk J, Panek D, Malawska B (2016). Therapeutic strategies for Alzheimer’s disease in clinical trials. Pharmacol Rep.

[5] Reitz C, Brayne C, Mayeux R (2011). Epidemiology of Alzheimer disease. Nat Rev Neurol.

[6] Dubois B, Feldman HH, Jacova C, Hampel H, Molinuevo JL, Blennow K, DeKosky ST, Gauthier S, Selkoe D, Bateman R (2014). Advancing research diagnostic criteria for Alzheimer’s disease: the IWG-2 criteria. Lancet Neurol.

[7] Reitz C, Mayeux R (2014). Alzheimer disease: epidemiology, diagnostic criteria, risk factors and biomarkers. Biochem Pharmacol.

[8] Graham WV, Bonito-Oliva A, Sakmar TP (2017). Update on Alzheimer’s disease Therapy and Prevention Strategies. Annu Rev Med.

[9] Grimm A, Mensah-Nyagan AG, Eckert A (2016). Alzheimer, mitochondria and gender. Neurosci Biobehav Rev.

[10] Cummings JL, Morstorf T, Zhong K (2014). Alzheimer’s disease drug-development pipeline: few candidates, frequent failures. Alzheimers Res Ther.

[11] Davies P, Maloney AJ (1976). Selective loss of central cholinergic neurons in Alzheimer’s disease. Lancet.

[12] Upton N, Chuang TT, Hunter AJ, Virley DJ (2008). 5-HT6 receptor antagonists as novel cognitive enhancing agents for Alzheimer’s disease. Neurotherapeutics.

[13] Brauser D: Two more phase 3 trials of Alzheimer’s drug idalopirdine fail. Medscape; 2017. http://www.medscape.com/viewarticle/875632.

[14] Axovant Unveils New Data Analysis Showing Addition of Intepirdine to Standard Therapy May Help People with Alzheimer’s Disease Maintain Independence Longer. PR Newswire; 2016. http://www.prnewswire.com/news-releases/axovant-unveils-new-data-analysis-showing-addition-of-intepirdine-to-standard-therapy-may-help-people-with-alzheimers-disease-maintain-independence-longer-300376136.html.

[15] Passani MB, Blandina P (1998). Cognitive implications for H3 and 5-HT3 receptor modulation of cortical cholinergic function: a parallel story. Methods Find Exp Clin Pharmacol.

[16] Haig GM, Pritchett Y, Meier A, Othman AA, Hall C, Gault LM, Lenz RA (2014). A randomized study of H3 antagonist ABT-288 in mild-to-moderate Alzheimer’s dementia. J Alzheimers Dis.

[17] Kubo M, Kishi T, Matsunaga S, Iwata N (2015). Histamine H3 receptor antagonists for Alzheimer’s disease: a systematic review and meta-analysis of randomized placebo-controlled trials. J Alzheimers Dis.

[18] Lombardo S, Maskos U (2015). Role of the nicotinic acetylcholine receptor in Alzheimer’s disease pathology and treatment. Neuropharmacology.

[19] Woodruff-Pak DS, Vogel RW, Wenk GL (2001). Galantamine: effect on nicotinic receptor binding, acetylcholinesterase inhibition, and learning. Proc Natl Acad Sci U S A.

[20] Russo P, Del Bufalo A, Frustaci A, Fini M, Cesario A (2014). Beyond acetylcholinesterase inhibitors for treating Alzheimer’s disease: alpha7-nAChR agonists in human clinical trials. Curr Pharm Des.

[21] Hung SY, Huang WP, Liou HC, Fu WM (2015). LC3 overexpression reduces Abeta neurotoxicity through increasing alpha7nAchR expression and autophagic activity in neurons and mice. Neuropharmacology.

[22] Hung SY, Huang WP, Liou HC, Fu WM (2009). Autophagy protects neuron from Abeta-induced cytotoxicity. Autophagy.

[23] Prickaerts J, van Goethem NP, Chesworth R, Shapiro G, Boess FG, Methfessel C, Reneerkens OA, Flood DG, Hilt D, Gawryl M (2012). EVP-6124, a novel and selective alpha7 nicotinic acetylcholine receptor partial agonist, improves memory performance by potentiating the acetylcholine response of alpha7 nicotinic acetylcholine receptors. Neuropharmacology.

[24] Rare but severe side effects sideline some phase 3 encenicline trials [http://www.alzforum.org/news/research-news/rare-severe-side-effects-sideline-some-phase-3-encenicline-trials].

[25] Hynd MR, Scott HL, Dodd PR (2004). Glutamate-mediated excitotoxicity and neurodegeneration in Alzheimer’s disease. Neurochem Int.

[26] Thomas SJ, Grossberg GT (2009). Memantine: a review of studies into its safety and efficacy in treating Alzheimer’s disease and other dementias. Clin Interv Aging.

[27] Doody RS, Tariot PN, Pfeiffer E, Olin JT, Graham SM (2007). Meta-analysis of six-month memantine trials in Alzheimer’s disease. Alzheimers Dement.

[28] Schneider LS, Dagerman KS, Higgins JP, McShane R (2011). Lack of evidence for the efficacy of memantine in mild Alzheimer disease. Arch Neurol.

[29] Wang SJ, Wang KY, Wang WC (2004). Mechanisms underlying the riluzole inhibition of glutamate release from rat cerebral cortex nerve terminals (synaptosomes). Neuroscience.

[30] Fumagalli E, Funicello M, Rauen T, Gobbi M, Mennini T (2008). Riluzole enhances the activity of glutamate transporters GLAST, GLT1 and EAAC1. Eur J Pharmacol.

[31] Grant P, Song JY, Swedo SE (2010). Review of the use of the glutamate antagonist riluzole in psychiatric disorders and a description of recent use in childhood obsessive-compulsive disorder. J Child Adolesc Psychopharmacol.

[32] Hunsberger HC, Weitzner DS, Rudy CC, Hickman JE, Libell EM, Speer RR, Gerhardt GA, Reed MN (2015). Riluzole rescues glutamate alterations, cognitive deficits, and tau pathology associated with P301L tau expression. J Neurochem.

[33] Selkoe DJ, Hardy J (2016). The amyloid hypothesis of Alzheimer’s disease at 25 years. EMBO Mol Med.

[34] Hardy J, Selkoe DJ (2002). The amyloid hypothesis of Alzheimer’s disease: progress and problems on the road to therapeutics. Science.

[35] Kandalepas PC, Sadleir KR, Eimer WA, Zhao J, Nicholson DA, Vassar R (2013). The Alzheimer’s beta-secretase BACE1 localizes to normal presynaptic terminals and to dystrophic presynaptic terminals surrounding amyloid plaques. Acta Neuropathol.

[36] Lilly Halts Phase 2 Trial of BACE inhibitor due to liver toxicity. 2013. http://www.alzforum.org/news/research-news/lilly-halts-phase-2-trial-bace-inhibitor-due-liver-toxicity.

[37] Kennedy ME, Stamford AW, Chen X, Cox K, Cumming JN, Dockendorf MF, Egan M, Ereshefsky L, Hodgson RA, Hyde LA (2016). The BACE1 inhibitor verubecestat (MK-8931) reduces CNS beta-amyloid in animal models and in Alzheimer’s disease patients. Sci Transl Med.

[38] Hawkes N (2017). Merck ends trial of potential Alzheimer’s drug verubecestat. BMJ.

[39] McCarthy JV, Twomey C, Wujek P (2009). Presenilin-dependent regulated intramembrane proteolysis and gamma-secretase activity. Cell Mol Life Sci.

[40] Doody RS, Raman R, Farlow M, Iwatsubo T, Vellas B, Joffe S, Kieburtz K, He F, Sun X, Thomas RG (2013). A phase 3 trial of semagacestat for treatment of Alzheimer’s disease. N Engl J Med.

[41] Bulic B, Ness J, Hahn S, Rennhack A, Jumpertz T, Weggen S (2011). chemical biology, molecular mechanism and clinical perspective of gamma-secretase modulators in Alzheimer’s disease. Curr Neuropharmacol.

[42] Lemere CA (2013). Immunotherapy for Alzheimer’s disease: hoops and hurdles. Mol Neurodegener.

[43] Schenk D, Barbour R, Dunn W, Gordon G, Grajeda H, Guido T, Hu K, Huang J, Johnson-Wood K, Khan K (1999). Immunization with amyloid-beta attenuates Alzheimer-disease-like pathology in the PDAPP mouse. Nature.

[44] Holmes C, Boche D, Wilkinson D, Yadegarfar G, Hopkins V, Bayer A, Jones RW, Bullock R, Love S, Neal JW (2008). Long-term effects of Abeta42 immunisation in Alzheimer’s disease: follow-up of a randomised, placebo-controlled phase I trial. Lancet.

[45] Sterner RM, Takahashi PY, Yu Ballard AC (2016). Active Vaccines for Alzheimer Disease Treatment. J Am Med Dir Assoc.

[46] Orgogozo JM, Gilman S, Dartigues JF, Laurent B, Puel M, Kirby LC, Jouanny P, Dubois B, Eisner L, Flitman S (2003). Subacute meningoencephalitis in a subset of patients with AD after Abeta42 immunization. Neurology.

[47] Winblad B, Andreasen N, Minthon L, Floesser A, Imbert G, Dumortier T, Maguire RP, Blennow K, Lundmark J, Staufenbiel M (2012). Safety, tolerability, and antibody response of active Abeta immunotherapy with CAD106 in patients with Alzheimer’s disease: randomised, double-blind, placebo-controlled, first-in-human study. Lancet Neurol.

[48] Arai H, Suzuki H, Yoshiyama T (2015). Vanutide cridificar and the QS-21 adjuvant in Japanese subjects with mild to moderate Alzheimer’s disease: results from two phase 2 studies. Curr Alzheimer Res.

[49] Davtyan H, Bacon A, Petrushina I, Zagorski K, Cribbs DH, Ghochikyan A, Agadjanyan MG (2014). Immunogenicity of DNA- and recombinant protein-based Alzheimer disease epitope vaccines. Hum Vaccin Immunother.

[50] Vandenberghe R, Rinne JO, Boada M, Katayama S, Scheltens P, Vellas B, Tuchman M, Gass A, Fiebach JB, Hill D (2016). Bapineuzumab for mild to moderate Alzheimer’s disease in two global, randomized, phase 3 trials. Alzheimers Res Ther.

[51] Abbott A, Dolgin E (2016). Failed Alzheimer’s trial does not kill leading theory of disease. Nature.

[52] Panza F, Solfrizzi V, Imbimbo BP, Logroscino G (2014). Amyloid-directed monoclonal antibodies for the treatment of Alzheimer’s disease: the point of no return?. Expert Opin Biol Ther.

[53] Panza F, Solfrizzi V, Imbimbo BP, Tortelli R, Santamato A, Logroscino G (2014). Amyloid-based immunotherapy for Alzheimer’s disease in the time of prevention trials: the way forward. Expert Rev Clin Immunol.

[54] Sevigny J, Chiao P, Bussiere T, Weinreb PH, Williams L, Maier M, Dunstan R, Salloway S, Chen T, Ling Y (2016). The antibody aducanumab reduces Abeta plaques in Alzheimer’s disease. Nature.

[55] Buee L, Bussiere T, Buee-Scherrer V, Delacourte A, Hof PR (2000). Tau protein isoforms, phosphorylation and role in neurodegenerative disorders. Brain Res Brain Res Rev.

[56] Lace GL, Wharton SB, Ince PG (2007). A brief history of tau: the evolving view of the microtubule-associated protein tau in neurodegenerative diseases. Clin Neuropathol.

[57] Panza F, Solfrizzi V, Seripa D, Imbimbo BP, Lozupone M, Santamato A, Zecca C, Barulli MR, Bellomo A, Pilotto A (2016). Tau-centric targets and drugs in clinical development for the treatment of Alzheimer’s disease. Biomed Res Int.

[58] Butler D, Bendiske J, Michaelis ML, Karanian DA, Bahr BA (2007). Microtubule-stabilizing agent prevents protein accumulation-induced loss of synaptic markers. Eur J Pharmacol.

[59] Wischik CM, Staff RT, Wischik DJ, Bentham P, Murray AD, Storey JM, Kook KA, Harrington CR (2015). Tau aggregation inhibitor therapy: an exploratory phase 2 study in mild or moderate Alzheimer’s disease. J Alzheimers Dis.

[60] Baddeley TC, McCaffrey J, Storey JM, Cheung JK, Melis V, Horsley D, Harrington CR, Wischik CM (2015). Complex disposition of methylthioninium redox forms determines efficacy in tau aggregation inhibitor therapy for Alzheimer’s disease. J Pharmacol Exp Ther.

[61] Panza F, Solfrizzi V, Seripa D, Imbimbo BP, Lozupone M, Santamato A, Zecca C, Barulli MR, Bellomo A, Pilotto A, et al: Tau-centric targets and drugs in clinical development for the treatment of Alzheimer’s disease. Biomed Res Int 2016. 10.1155/2016/3245935 PMC4939203 27429978

[62] Gruninger F (2015). Invited review: drug development for tauopathies. Neuropathol Appl Neurobiol.

[63] Theunis C, Crespo-Biel N, Gafner V, Pihlgren M, Lopez-Deber MP, Reis P, Hickman DT, Adolfsson O, Chuard N, Ndao DM (2013). Efficacy and safety of a liposome-based vaccine against protein Tau, assessed in tau.P301L mice that model tauopathy. PLoS One.

[64] Aisen PS, Gauthier S, Ferris SH, Saumier D, Haine D, Garceau D, Duong A, Suhy J, Oh J, Lau WC, Sampalis J (2011). Tramiprosate in mild-to-moderate Alzheimer’s disease - a randomized, double-blind, placebo-controlled, multi-centre study (the Alphase Study). Arch Med Sci.

[65] Jaturapatporn D, Isaac MG, McCleery J, Tabet N: Aspirin, steroidal and non-steroidal anti-inflammatory drugs for the treatment of Alzheimer’s disease. Cochrane Database Syst Rev. 2012;(2):CD006378. doi:10.1002/14651858.CD006378.pub2. https://www.ncbi.nlm.nih.gov/pubmed/22336816. 10.1002/14651858.CD006378.pub2 PMC11337172 22336816

[66] Porrini V, Lanzillotta A, Branca C, Benarese M, Parrella E, Lorenzini L, Calza L, Flaibani R, Spano PF, Imbimbo BP, Pizzi M (2015). CHF5074 (CSP-1103) induces microglia alternative activation in plaque-free Tg2576 mice and primary glial cultures exposed to beta-amyloid. Neuroscience.

[67] Kaveri SV (2012). Intravenous immunoglobulin: exploiting the potential of natural antibodies. Autoimmun Rev.

[68] Dodel R, Hampel H, Depboylu C, Lin S, Gao F, Schock S, Jackel S, Wei X, Buerger K, Hoft C (2002). Human antibodies against amyloid beta peptide: a potential treatment for Alzheimer’s disease. Ann Neurol.

[69] Relkin NR, Thomas RG, Rissman RA, Brewer JB, Rafii MS, van Dyck CH, Jack CR, Sano M, Knopman DS, Raman R (2017). A phase 3 trial of IV immunoglobulin for Alzheimer disease. Neurology.

[70] Frolich L, Blum-Degen D, Riederer P, Hoyer S (1999). A disturbance in the neuronal insulin receptor signal transduction in sporadic Alzheimer’s disease. Ann N Y Acad Sci.

[71] Reger MA, Watson GS, Green PS, Wilkinson CW, Baker LD, Cholerton B, Fishel MA, Plymate SR, Breitner JC, DeGroodt W (2008). Intranasal insulin improves cognition and modulates beta-amyloid in early AD. Neurology.

[72] Paris D, Bachmeier C, Patel N, Quadros A, Volmar CH, Laporte V, Ganey J, Beaulieu-Abdelahad D, Ait-Ghezala G, Crawford F, Mullan MJ (2011). Selective antihypertensive dihydropyridines lower Abeta accumulation by targeting both the production and the clearance of Abeta across the blood-brain barrier. Mol Med.

[73] Lawlor B, Kennelly S, O'Dwyer S, Cregg F, Walsh C, Coen R, Kenny RA, Howard R, Murphy C, Adams J (2014). NILVAD protocol: a European multicentre double-blind placebo-controlled trial of nilvadipine in mild-to-moderate Alzheimer’s disease. BMJ Open.

[74] Prashad S, Filbey FM (2017). Cognitive motor deficits in cannabis users. Curr Opin Behav Sci.

[75] Cummings J, Aisen PS, DuBois B, Frolich L, Jack CR, Jones RW, Morris JC, Raskin J, Dowsett SA, Scheltens P: Drug development in Alzheimer’s disease: the path to 2025. Alzheimers Res Ther. 2014;6(4):37. doi:10.1186/alzrt269. eCollection 2014. https://www.ncbi.nlm.nih.gov/pubmed/25024750. 10.1186/s13195-016-0207-9 PMC5028936 27646601

